# Strategies for data analytics projects in business performance forecasting: a field study

**DOI:** 10.1007/s00187-022-00338-7

**Published:** 2022-04-04

**Authors:** Maël Schnegg, Klaus Möller

**Affiliations:** University of St. Gallen, St. Gallen, Switzerland

**Keywords:** Data analytics, Performance, Forecasting, Field study

## Abstract

Data analytics is applied in various fields, including business performance forecasting, but companies struggle with its implementation. Following a cross-sectional field study approach, we make two contributions. First, we elaborate on the central role played by the head controller in generating trust in analytics solutions and thus, making the project successful. Second, we identify three patterns in the way companies plan, implement, and then use data analytics in the context of business performance forecasting. The two successful patterns are the ones that start with a limited but tangible objective (either in term of information precision, or rapidity of processing) that can be expended in a second time.

## Introduction

The objective of managers is for their organizations to perform well and grow. To achieve this aim, they implement systems to control whether the strategy is realized effectively, resulting in increased performance and growth (Bedford, 2015; De Geuser et al., 2009; Simons, 1990). Various management control systems have been developed to drive firms’ performance (Malmi & Brown, 2008; Otley, 1994; Simons, 1994, 1995). The forecasting procedure, on which this paper focuses, is an important part of such a system. Precise forecasting allows managers to shift and allocate resources in anticipation to optimally meet business needs. A company’s success, therefore, depends on how well it can forecast and, consequently, adapt its business operations and its strategy (Rohrbeck & Kum, 2018). From a constructivist perspective, the completeness of forecasting systems’ prediction is limited (Rossel, 2012), which is particularly true in an uncertain environment (Hansen et al., 2003; Otley, 2016).

Further, researchers increasingly investigate whether and how their forecasts’ accuracy and efficiency are, in fact, improving (Dhaliwal et al., 2012; Easterwood & Nutt, 1999; Liu & Natarajan, 2012). Currently, forecasts lack precision and cover a shorter horizon when business environment uncertainty increases (Peter & Jarratt, 2014; Vecchiato, 2012a, 2012b). Such an evolution of business forecasting generates a need for novel ways to develop forecasts, and one promising approach is to rely on digitalization and, more specifically, data analytics.

The digitalization, among others, of management control systems has quite radically modified their design and use over the last decades (Chenhall & Moers, 2015) and has received increasing attention (Quattrone, 2016). Forecasting is amongst the first management control systems to obtain inputs from data analytics (Bergmann et al., 2020). Further, during the last decade, two major technological data developments could potentially impact the use of management control systems. First, technology, such as in-memory data storage, which can cope with large datasets, is now available (Neumann et al., 2012; Wilson & Demers, 2015). Second, digitization has allowed a vast amount of data to be accumulated at a constantly increasing pace, which is now available for analysis (Changala & Rajeswara Rao, 2017). These changes have led to increased data analytics applications in various areas (Fayyad & Uthurusamy, 2002). For example, data analytics in the form of data mining has become a popular instrument for forecasting problems in the supply chain, macroeconomics, and financial markets fields (Bansal et al., 2009; Olson & Wu, 2017), which technology experts promoted as “predictive analytics” (Davenport, 2006). Consequently, three positive performance management outcomes use data analytics: increased efficiency, accuracy, and a better understanding of the business drivers (Roßmann et al., 2018; Wang et al., 2018; Wilson, 2018).

However, strong empirical evidence is still lacking in the literature on the potential use of data analytics. Various calls have been made for data analytics’ current status in firms to be better investigated and for the proposed models to be tested (Gärtner & Hiebl, 2017; Rikhardsson & Yigitbasioglu, 2018).

A new system’s implementation process implies many potential changes, both during the process, which could be time-consuming (Holland & Light, 1999) and after it, which could permanently alter the managerial and business processes. The latter might happen because “legacy systems encapsulate the existing business processes, organization structure, culture, and information technology” (Holland & Light, 1999, 91). In other words, existing systems reflect contingency variables (Otley, 2016). Therefore, this study will examine the implementation process from the business need up to the use of the data analytical tool supporting the forecasting process.

Early on in the development of analytics techniques, financial data was one area that merited particular interest (Chen et al., 2012), particularly in accounting. If initially, the focus was on opportunities to automate certain processes such as auditing (Cao et al., 2015; Yoon et al., 2015), the discussion rapidly broadened to the speed at which data can be analyzed, impacting budgeting processes (LaValle et al., 2011; Warren et al., 2015). This also added the debate on moving towards “beyond budgeting” and relying on technologies to move out of certain static practices (Bergmann et al., 2020; Warren et al., 2015). Having reached a certain maturity in terms of diffusion and use in the financial function makes analytics an interesting candidate to investigate the drivers of the use of a technology to achieve a variety of objectives.

Managers can implement various technical solutions to take advantage of management control systems’ digitalization. One of them is data analytics. It is used to “describe the data sets and analytical techniques in applications that are so large (from terabytes to exabytes) and complex (from sensors to social media data) that they require advanced and unique data storage, management, analysis, and visualization technologies” (Chen et al., 2012). Big data is a term used to illustrate the reliance on a large quantity and diversity of data (Chen et al., 2012; Sledgianowski et al., 2017). The concept of data analytics also integrates the managerial purpose behind big sets of data which is to help the decision process (Chen et al., 2012; Schneider et al., 2015). Forecasts are developed to help in the decision -process. For the sake of clarity, we use the term data analytics, which is more inclusive and encompasses managerial and analytical capabilities.

Data analytics has been discussed extensively in practice and in some research fields. Despite its potential, however, data analytics is not yet widely applied to forecast business performance. A recent practitioner study suggests that only 21% of companies use data analytics in the context of management accounting (Grönke et al., 2018). Our paper studies some of the reasons that can explain a rather low rate of adoption despite the promises of data analytics. Examples of failed implementation and the fear of failure are preventing the deployment of data analytics (Larson & Chang, 2016; Wang et al., 2018). Consequently, this paper aims to enhance our understanding of the success factors of data analytics’ implementation in the management accounting context and, more specifically, in the context of business performance forecasting. This objective raises the following research question: What are the conditions and patterns that make a data analytics project successful in terms of business performance forecasting? Observations showed the following results: two factors (individually and combined) appear to be prominent. They are in line with and contribute to previous research. The first success factor is the capacity to set clear goals for data analytics with regards to process efficiency, output accuracy and explainability, before the project starts (Wang et al., 2018). The second one relies on the capability of the controller to take a leadership role in the project (Wolf et al., 2020). Extant literature already discusses the role of controller and the need to enlarge the scope of his responsibilities (Goretzki et al., 2013). Our observations show that this might be of particular importance in data analytics projects.

We explore the phenomenon by following a cross-sectional field study approach (Lillis & Mundy, 2005). The rest of the paper is structured as follows: the next Sect. 2 reviews the literature by examining the field of forecasting and performance management. We elaborate on the methodology in Sect. 3. Section 4 presents the six cases and provide insights into the interviews held at the various companies. Section 5 presents our findings. The latter is twofold: first, we review the role of controllers leading such projects. In the second part of the findings, we propose a framework for categorizing these projects. Section 6, the last one, provides a conclusion and an outlook for future research.

## Literature review

The introduction shed light on the current limited use of data analytics tools in organizations (Grönke et al., 2018). The finance department of firms usually hosts a vast amount of data which can potentially be used in decision-making processes (Chen et al., 2012). Moreover, it has been suggested that data analytics could not only help at better leveraging the available data but also in changing the way some controlling process, in particular, budgeting and forecasting, unfold in organizations and how controllers can support these changes (Cao et al., 2015; Yoon et al., 2015).

Consequently, the initial part in the literature review concerns the use of data analytics in the specific field of management accounting. The existing and potential uses are covered here.

### Data analytics and management accounting

Enhancing control systems with advanced data analytics (and big data) is a recent but growing development (Leon et al., 2012; Schneider et al., 2015). Nevertheless, it is unclear how the latter development could renew or modify the way accounting is performed and understood or what the managerial impact of this change could be (Bhimani & Willcocks, 2014; Griffin & Wright, 2015). Some researchers regard such a change as a total game-changer (Appelbaum et al., 2017; Vasarhelyi et al., 2015) that could alter the way firms are controlled permanently and profoundly, as well as the way they need to communicate their performance. For example, some researchers hypothesize that this change could be the end of static accounting (Krahel & Titera, 2015).

Other researchers consider data analytics as a new technology that could have a disruptive impact in the short run but ultimately merely improve current processes (Quattrone, 2016) by leveraging on automation (analytical tools) and relying on more data (big data). The situation could be compared to the arrival of the first Information Systems, such as ERP (Alles, 2015). In his essay, Quattrone (2016) expresses the fear that data analytics might have counterproductive results: “If I had to bet on what big data will do for decision-making,^1^ I would say that it will make people take wrong decisions much more quickly than before, with even less room for the exercise of wisdom beyond the increasing compliance that affects various realms of decision-making, from finance to risk management.” Although many studies emphasize predictive analytics’ potential for companies in terms of their efficiency, accuracy, and business insights, there is no actual evidence of the results (Shmueli & Koppius, 2010). The extent to which accounting, and finance function could benefit from data analytics is still unclear.

Conceptually, the literature provides studies on data analytics’ potential impacts on the accounting function, management accounting practices and, very specifically, on business forecasting (Appelbaum et al., 2017; Bhimani & Willcocks, 2014; Cao et al., 2015; Schneider et al., 2015). Other scholars advocate for more empirical research (Gärtner & Hiebl, 2017; Rikhardsson & Yigitbasioglu, 2018). In a special issue of the Journal of Management Control, the editors suggest that the digitalization of the finance and accounting function might not be management boards’ top priority. Consequently, the number of cases where data analytics is applied in accounting is still limited, as the implementation is still in progress (Möller et al., 2020). Therefore, this paper aims to address the observed gap in the empirical research on data analytics in terms of the accounting function.

In the same special issue, four empirical research studies investigate the application of data analytics in accounting using various methods (Bergmann et al., 2020; Knauer et al., 2020; Perkhofer et al., 2020; Vitale et al., 2020). Bergmann et al. (2020) specifically study the application of business analytics in the context of budgeting. They justify their choice by maintaining that digitalization’s expected results can already be easily observed and even be measured. According to these scholars, digitalization’s first impact concerned time saving, while the second is the increased convenience when budgeting. Bergmann et al. (2020) also regard budgeting as probably the first to be digitalized because this process is critical and of utmost importance for most companies. If the budgeting process could significantly impact the overall organization, a minimal number of people are usually allowed access to it. Nevertheless, Bergmann et al.’s (2020) paper focuses on digitalization’s technical aspects (data infrastructure) and other determinants, which might need to be investigated too.

The future use of analytics in the finance function is highly dependent on firms’ capabilities to implement these new analytical tools. Various challenges associated with the use of data analytics and management accounting have been identified (Gärtner & Hiebl, 2017). Responding successfully to them can increase the success rate of implementations. Bergmann et al. (2020) investigate some conditions that can favor (or prevent) the use of analytical tools in the budgeting function. To better investigate the specific potential success factors in the implementation of data analytics for business forecasting, it is necessary to investigate what was suggested in the general information systems literature regarding the success and failure factors in project implementation. This is discussed in the second part of the literature review.

### Success factors and information systems

Information systems can be considered the generic research field to which data analytics belong and within it, a specific stream focused on the evaluation of the success factors of information systems. This evaluation has a long history, starting with its earliest implementations (Bullen & Rockart, 1981; Daniel, 1961). Here, there have been many contributions, including identifying the success factors of specific systems. One challenge of the field concerns the joint effects (e.g., two factors in conjunction could moderate their individual effects) of certain factors but many of the systems and factors overlap (Biehl, 2007; Holland & Light, 1999; Poon & Wagner, 2001). Since data analytics is also part of the information system field, these contributions could also serve to evaluate data analytics’ success factors. In addition, there are also contributions studying data analytics in management control systems (Möller et al., 2016) where management control systems are defined as “formal, information-based routines and procedures managers use to maintain or alter patterns in organizational activities” (Simons, 1995).

Researchers have tried to identify success factors by using a broad set of methodologies. These methodologies range from field research, such as case studies (Yeoh & Koronios, 2010), to survey-like methods (Pinto & Slevin, 1987), to systematic literature reviews (DeLone & McLean, 1992). Once again, using multiple methodologies, researchers’ propositions are also constantly refined (Seddon, 1997; Wu & Wang, 2006). These researchers have built frameworks that help categorize and identify the relevant success factors. While some focus on technical aspects such as data or models (Severtson et al., 2017; Wirth & Hipp, 2000). They also question the way it is implemented (Klatt et al., 2011; Schläfke et al., 2013).

The potential success factors vary due to the great diversity of factors, such as the people implementing and using the system, the implementing partners or the people within the organization in charge of the project (Antoniadis et al., 2015; Ram et al., 2014). People are not the only factor that is identified, the technologies and software selected play an important role (Bansal et al., 2009; Reitsma et al., 2018). The resources dedicated (time, employees) and the general level of dedication will significantly facilitate or impede the successful implementation of an information system (Reitsma et al., 2018). Another set of factors relates to the organization’s starting point in terms of digitalization, culture, and the existing system that can favor the development of a project (Raafat Saade & Nijherhors, 2016) or the problem that the system aims to solve. More importantly, these factors are often studied independently, but some researchers confirmed the need to investigate the interactions of their different effects (Bansal & Agarwal, 2015).

Various aspects related to the people involved in the development of a project (Acuña & Juristo, 2004) have been identified to play a role in the success of the implementation such as the trust between the people (Antoniadis et al., 2015), the capabilities of the people to learn or lead a project (Ram et al., 2014) and to communicate with other stakeholders (Raafat Saade & Nijherhors, 2016). The ability of certain people to fulfill a specific role (Byrne & Pierce, 2007; Wolf et al., 2020) in the context of the project is of great importance and it is the subject of the third part of our literature review.

### Roles in information systems project management

Individual characteristics of managers have been considered to impact the implementation process of management control systems and financial practices (Morelli & Lecci, 2014; Plöckinger et al., 2016). The literature on information systems has also considered these factors as influencing the implementation success of information systems (Bansal & Agarwal, 2015; Ram et al., 2014). These studies on the success factors for information systems’ implementation mentioned a few different stakeholders involved in the process. For example, the top management team and the support it can bring to the project, the external supplier (vendor of the software), and the project team (Bansal & Agarwal, 2015; Raafat Saade & Nijherhors, 2016; Reitsma et al., 2018).

Each stakeholder will assume a certain role, determined by his capabilities, the specific context (company structure, size, etc.), and the expectations of the people he is interacting with (Byrne & Pierce, 2007). These determinants can evolve and so the initial role of the incumbent might also have to adapt (Acuña & Juristo, 2004).

The role in the accounting and finance function is of particular interest in the cases studied in this paper, as the six data analytics implementation projects were related to the accounting function, and more precisely, forecasting. It was shown that CFOs’ antecedents and capabilities could have a major impact on the decision to implement and the success of an ERP deployment (Acuña & Juristo, 2004; Hiebl et al., 2017). In fact, a CFO’s understanding and beliefs of the software industry can play a significant role in such projects too (Lepistö, 2014). Sometimes described as performing a rather mechanical task, controllers can take multiple roles to fit with a specific situation, such as coordination and support (Goretzki et al., 2013; Unger et al., 2012; Wolf et al., 2020). Consequently, they can play the role of catalyst to create a coalition around a specific project (Chua et al., 2012). Regarding innovative projects in data analytics, controllers are also recognized to positively impact the success of a project in situations where uncertainty need to be processed is high as in such situation the need for processing knowledge (to reduce uncertainty) is also high (Malagueño et al., 2021).

There are many open questions as to how data analytics will shape the future of management accounting and the role of controllers. Taking into account the potentially radical changes for the controlling function, the paper aims to study how the success factors identified for other types of projects (e.g., ERP implementation) hold with data analytics as well as how the current and existing role of controller impact project success and how the aspired role of controller could favor data analytics implementation in the future.

## Methodology

Scholars often suggest using a qualitative approach to explore a new phenomenon such as data analytics (Berg & Lune, 2001; Eisenhardt, 1989; Eisenhardt & Graebner, 2007). Various theoretical and conceptual papers on data analytics in management accounting have indeed been published (Gärtner & Hiebl, 2017; Rikhardsson & Yigitbasioglu, 2018; Vasarhelyi et al., 2015), but there are very few empirical papers on these topics (Bergmann et al., 2020; Möller et al., 2020). We, therefore, regard cross-sectional field studies and case studies as appropriate methodologies for such research.

Specifically, we used cross-sectional field studies as our research methodology (Ittner & Larcker, 2001; Lillis & Mundy, 2005; Merchant & Manzoni, 1989; Reinking et al., 2020). On the one hand, our objective was to study multiple cases to ensure diversity and offer a broad enough perspective on the topic while benefitting from the qualitative research approach’s flexibility, which a survey might not offer, given our research’s exploratory nature. In terms of data analytics and especially its application in management accounting (and budgeting), our methodology allows for a qualitative field study, which provides the opportunity to advance general propositions based on multiple observations. At present, it is still difficult to identify a sufficient number of completed data analytics projects with either a positive or negative result. Furthermore, the novelty of the field complicates achieving a common understanding and arriving at shared definitions. Our semi-structured interviews and discussions helped us create a shared understanding of the topic, thus ensuring a balanced view of the various discussed cases.

We also relied on the methodology literature on case study building when selecting cases (Berg & Lune, 2001; Cooper & Morgan, 2008; Eisenhardt & Graebner, 2007; Gerring, 2006; Mikes, 2011). Various targets were set. First, the cases had to apply data analytics, and the project had to be sufficiently advanced to understand its potential failure or success factors. Furthermore, all the projects had to apply data analytics for forecasting. As previously explained, forecasting is an appropriate candidate for using data analytics. Selecting cases with one similar application prevents our results suffering from other unidentified effects. Second, the data application had to be in a management accounting context due to our selecting forecasting activities. Third, we focused on medium to large companies since small companies do not usually have the means to implement adapted or customized solutions. Instead, as various practical studies^2^ have shown, they use out-of-the-box solutions with limited capabilities. Fourth, since our objective was to identify success and/or failure factors, both successful and unsuccessful implementations had to be present in the sample cases. Fifth, the interviewees’ diversity was also important. Since these people worked at the intersection between data analytics and management accounting, it was important to gain an understanding of the scientists in charge of the technical solution and that of the managers in charge of distributing the solution throughout the organization. The diverse profiles also helped better understand the role of each stakeholder and the perceptions that others had of them. Sixth, it was important that the case selection included a diversity of industry, facilitating the results’ generalization.

Given the above criteria and the limited number of companies currently applying data analytics when forecasting their business performance, it was challenging to find matching companies and suitable interview partners.

We chose a semi-structured interview (see appendix 1—Interview Guideline) to collect data. Using an interview guide directs the interview according to the developed framework. Still, it leaves the interviewees with enough scope to address aspects that the author, who followed the narrowly formulated questions, might not have potentially covered (Berg & Lune, 2001). The objective of the interview guidelines was to capture sufficiently broad perspectives. It was built upon previously suggested categories (Möller et al., 2016; Pinto & Slevin, 1987; Yeoh & Koronios, 2010), giving sufficient confidence that we would capture diverse signals pointing toward where companies should be focusing their efforts, as shown in Sect. 4. All the interviews were carried out by a native German speaker and lasted approximately an hour each. They were recorded and transcribed. Some of them were recorded in German and, after that, translated into English. A neutral researcher, not the interviewer, was responsible for translating the relevant interviews. The analysis was done by placing the German transcripts and their English translation side by side to avoid their meaning being distorted during the analysis process (Feldermann & Hiebl, 2019). The interviews were complemented with additional data, such as press articles.

## Case studies

This section presents the six selected case studies and the interviewees’ observations about their respective projects. Table 1 presents an overview of the case studies.

**Table 1:** Case studies overview

Company	Industry	Revenues (FY 17 in bn)	Use case	Interviewee	Success
TelCo	Telecom	EUR 75	Digital revenue forecast	Group controlling representatives (2)	No
ChemCo	Chemical	EUR 65	Machine-based forecast	Head of corporate controlling	Yes
FoodCo	Food anmd beverages	EUR + 50	Analytic sales forecast in R&D controlling	Project leader and data scientist	Open
TechCo	Semi-conductor	EUR 7	Short term sales forecast	Senior manager operation planning	Yes
SoftCo	Software	EUR 23	Travel expense forecast	Project leader, enterprise analytics	Yes
InduCo	Industrial	EUR 28	Analytic order entry forecast	Lead data scientist	No*

*Initially successful, the project was discontinued due to lack of financing

In Euro or equivalent at year end 2017.

Two interviewees participated in the TelCo case, while only a single interviewee participated in the other five cases. This is one of the study’s limitations, as interviewees’ views might not reflect those of the other stakeholders or the organization. There are various reasons for this limited number of interviews: first, there were only a few direct stakeholders fully involved in the forecasting project studied. Second, external stakeholders often help build the technical part of such a project. Third, data analytics is at an early stage of its development and implementation in companies, which might therefore be more reluctant to share information in this regard. Consequently, the diversity of the interviewees’ views (a technical versus business perspective) across the six cases was ensured in order to partially control for this limitation. As pointed out in the literature review, there is a critical need for empirical research even though the application of data analytics in management accounting is still at an early stage (Gärtner & Hiebl, 2017; Rikhardsson & Yigitbasioglu, 2018).

The following subsections will cover the various challenges identified in the literature review and report the interviewees’ most salient observations. As previously mentioned, the framework used for the interview (Möller et al., 2016) was designed to elicit the widest views of a data analytics project and helped categorize the information gained. We will present the observations in a similar structure as the one in which they were collected.

### TelCo

TelCo is a large telecommunication service provider based in Europe whose total sales exceed EUR 70 billion. We interviewed two of this company’s Group Controlling Representatives. Although TelCo’s existing forecasting process proved to be efficient and its prediction accuracy was adequate, its corporate controlling had a vision of using big data to produce a fully automated forecast. Corporate Controlling also expected the project to provide new business insights into what precisely drove their business.

According to the two controllers, the company’s first and biggest challenge was in terms of data, more specifically, the ease with which they could obtain these and the quality thereof (as well as the reprocessing they required). A representative of Group Controlling summarized the problem:

The one problem was the data. And specifically, the re-aggregation of the data.

He later added:

As I said, the crucial topic was how to reconcile the financial data and key figures from the operating data, (…) and how to have the local organization accept them.

Once obtained, Corporate Controlling had to process the data. Since the relevant employees did not know the specific statistical techniques to do so, they used a trial-and-error method, trying various statistical models to select the best fitting one. One of the Group Controlling representatives explained:

We tried a whole lot, that’s the nice thing in modern statistics, if you have a data cuboid in your system, you can try a large set of methods, and then decide at the end of the day for the best possible.

The lack of trust between the controllers and the top management team was a critical aspect:

The cooperation was not bad. It did not go in the direction of the top management team not wanting to work with us, but as my colleague said straight away, the lack of effort to drive the project was a hindrance. It was very close to the actual process, but they questioned its added value. You could describe it that way.

This lack of trust was overlooked for a long time. However, toward the end of the project, it became clear that this was undermining its success. The controllers—the two interviewees who led the project – perceived that the top management team, the team they considered responsible for driving the project, were not sufficiently involved. The two controllers assumed the roles of implementors. They proposed a viable solution to the top management. However, they were unable to lead the project and thus convince the top management of their prototype solution’s merits, also their stakeholders perceived the results as opaque. Consequently, the project was discontinued.

### ChemCo

ChemCo is a European chemical leader with revenues of more than EUR 60 billion per year. We interviewed the head of Corporate Controlling, who was responsible for the project. Owing to the individual business segments’ volatility, ChemCo made a yearly forecast, as well as one each month for the next six months. More than 1,000 employees were involved in the mostly manual process, gathering, and aggregating financial figures for the decentralized controlling organization each month. ChemCo formulated a digitization strategy involving a cut of 30 to 50% of the finance workforce within the next ten years, which was communicated. Consequently, ChemCo’s predictive analytics forecasting projects were mainly aimed at making the process more efficient.

Although the above might be a limitation of our study, its scope was reduced to available and identifiable data. According to the head of Corporate Controlling:

The data are mostly correct, but they are not properly allocated between the markets, sales units, and products groups. So, they classify it sometimes differently. For example, renovation, innovation, and normal sales. Here, I apply an algorithm to align them. But that’s about it. Since we use sales data, they are pretty clean.

However, the various stakeholders perceived the processing of the data as quite opaque, and a significant effort was required to make results as readable and transparent as possible:

We visualize the reconciliation between the divisions Forecast and our Predictive Forecast as being a front-end that compares the two forecasts. We also built a solution in Tableau showing in which month the results were driven most, also of the KPIs and the specific month, how strong the different effects were (…) Previously that had been a rather black box, but we have converted it into a grey box, which results in high forecast accuracy compared to that which the divisions themselves provide, as well as to a higher acceptance of the tools.

The project manager (the head of Corporate Control) was aware of the trust challenge and formally organized the transition period to not constrain the stakeholders. He took a leadership role, and the top management team let him organize the project how he perceived it was the most effective. They also maintained legacy and data analytics in parallel. Predictive Forecast’s representatives also made the precision gained through the new statistical methods visible in order to obtain the organization’s buy-in:

In 2017 we tracked our automatic forecast in parallel with that of the Forecast division, which led to the realization that we were usually more accurate than any other single division and in the group as a whole. Consequently, from 2018 we will be much clearer about this machine forecast (…) In 2019 we plan to turn the logic around and no longer ask the areas for their forecasts but use the machine predictions as the basis for our forecast, only asking the divisions to report deviations and unexpected events.

After that, the top management team was convinced of the need to digitalize the organization. This helped convince the various stakeholders and ensure that the financing would be available. The Head of Corporate Controlling explained:

We had a relatively strong push around digitization at ChemCo and ensured that everyone knew how important this digitization was through direct verbal communication, as well as permanent internal communication. Its importance is very high.

He later added:

We do not need top management support for this. I report to the CFO of the ChemCo Group, and the former CEO was also very open to the project. We did not get any push from them, but also no headwind.

These two quotes were quite instructive. The top management team support did not have to be direct in terms of the project, but rather in the freedom they allowed their employees.

Various stakeholders were invited to participate in the discussions early on to strengthen the project and facilitate the system’s adoption:

In the sense of change management, there is a regular exchange between the areas and discussions of the forecast’s status and further improvement. In other words, we try to involve the divisions in this process through regular discussions.

### FoodCo

FoodCo is a leading food and beverage company headquartered in Europe. FoodCo’s total sales exceed EUR 50 billion per year. We interviewed a senior predictive manager in the R&D department, who had led the development of a technical solution. FoodCo’s R&D controlling department started experimenting with a dynamic analytical forecasting tool for predicting the innovation and renovation pipeline’s sales per market segment and product category to improve the accuracy of its manually prepared forecasts. More accurate sales forecasts might also help allocate R&D investments where they were the most promising.

Digitalization and, more specifically, data analytics might generate resistance or tensions in organizations when they are first initiated. Consequently, FoodCo’s first major challenge was to ensure the transparency of the method used to analyze the data as reported by a Senior Predictive Manager:

“The challenge was their lack of understanding, which was understandable, because it [data analytics] was not their bread and butter, and they would have to let go of the steering wheel. They also felt that only humans can understand the market dynamics and make a prediction.

If there was a lack of trust and understanding, the people involved did not always clearly identify and express this. The problem was not identified before a certain period. Such an issue can result either from the users who did not share their concerns and consequently they would also not make the required effort to ensure the project was on track. They could also result from the implementor who was not attentive enough. If the Senior Predictive Manager briefly discussed about his customers (the marketing department) and the top managers during the interview, he described his project as being an IT challenge and merely identified other stakeholders such as controllers.

Changes were implemented gradually to overcome this challenge, avoid blockages, and create trust one step at a time. Here, the interviewee explains how changes were implemented:

It’s done via prototyping in small teams, which is what I am currently doing. Once something has been proven to work, a project is launched.

Consequently, the FoodCo data scientist did not need the top management’s constant full support:

I receive support in the sense that they allow me to work on it and develop this. This work is a recognized need, but for the rest I receive no support. Time is all I need. In the next steps, however, I will need more management support when it comes to convincing the business units.

However, a lack of sponsorship could alter the scope or at least slow down the project. At worst, this could indicate a total lack of management confidence, which could ultimately lead to the overall project being abandoned.

### TechCo

TechCo is a semi-conductor manufacturer based in Europe. The company reported revenues of more than EUR 7 billion in 2017. We discussed digitalization with the senior manager of Operations Planning. He led various company digitalization projects, all related to forecasts that could align sales and production better. Sales forecasts are crucial for supply chain operations. Due to the high cost of capital and their complex manufacturing processes, semi-conductors’ production sites are usually operated at full capacity. Incorrect forecasts might increase the unused capacities’ costs or lead to a loss of orders due to the manufacturer’s inability to deliver (Möller et al., 2016). The project was thus aimed at increasing the reliability of Operations Planning’s predictions.

TechCo’s data analytics project mainly focused on financial data, although this did not solve all the challenges. The senior manager of Operation Planning explained:

Technically, the biggest challenge was to ensure that the historical data were correct. We wanted to go back five years and for example, we had experienced a merger two and a half years ago. That was a big change. Then there was the question: do we have the previous data, and can we even compare them to today’s data? Consequently, continuity when just using quarterly figures is already a bit difficult. If I want to do statistics, I would like at least 100 points, but 25 years ago, our company did not yet exist. Five years back gives you 20 points, which is statistically questionable given that things change a lot in five years.

The legacy and data analytics forecasts were run in parallel to generate trust in the machine forecast:

As of today, this is just an additive signal that we provide (…) The crawl charts are simply an extra neutral signal, because sometimes you do not exactly know how up-to-date these forecasting activities are.

From a human resource and knowledge perspective, bringing the right people into the project was rather complicated:

The solutions these people needed to provide are rather isolated. For example, some IT people are more active in terms of projects. Then there is my function: I have been in finance since the middle of the year, and I deal with the drivers (…) In finance, something like a digital finance office needs to be set up, which should deal with such topics in terms of their finance. But IT also needs such an office. The landscape is therefore rather fragmented. The company does have approaches, but they have not as yet been equally formalized.

To be established in the long run and expanded, the Senior Manager at TechCo mentioned the need to create ownership and responsibilities within the finance department. The interviewed senior manager of Operation Planning explained the top management’s lack of interest when he started data analytics project for business forecasting:

We did not have strong management support. Consequently, we were self-motivated, starting with a student’s master thesis, which was actually just a test application. And that now has followers (…) I would say that the management support was moderate. We had to do a bit of persuasion, which we partially managed to do.

More generally, he also recognized that the implementation plan was not initially clear:

It was not systematic (…) But that was perhaps a weakness, perhaps we could have done something else.

Despite the top management’s poor support and the various stakeholders’ mild interest, the project manager (the senior manager of Operation Planning) got the project up and running. There were two main reasons for this: first, from his perspective (that of the data users), the need for the project was identified, which was the potential value-added. To overcome the lack of top support, the project was implemented gradually, and a great effort was made to convince the other stakeholders of its merit.

### SoftCo

SoftCo is a global software company headquartered in Europe. Besides its core business, SoftCo is also a leader in predictive analytics. The company’s total sales annually exceed EUR 20 billion. An enterprise analytics project leader was interviewed. SoftCo controllers produced financial forecasts manually and used Excel to calculate their projected figures. This forecasting process requires resources and takes a long time. An internal enterprise analytics department, which also serves as a predictive analytics software vendor, was also tasked with pushing the predictive use cases for internal purposes. The interviewee was selected to apply the use cases in the company.

The project that the enterprise analytics department had developed required the controllers to be onboarded early on to allow them to decide when the data analytics solution could go live. The project developers provided the controllers with easy-to-read dashboards, which they could use or not:

We also built a small dashboard to which the results are sent, and different measures control these results. We therefore have statistical metrics in it. The questions are: what are the deviations from the budget, and from the actuals? How are the Mean Average Percentage Error and other metrics displayed? If the controller with the predictive is satisfied, you can enter the data into the live version.

In the implementation phase, the controllers run the predictive analytics models:

We can have several versions running simultaneously and can also compare them. The controllers prepare the predictive run.

A significant effort was made to integrate the employees into the project and secure their trust:

That’s one of the most important topics. Predictive analytics is always great, and data scientists can create and render the best algorithms. Ultimately, however, it [the project] will fail because people say that they cannot be bothered, as they prefer to do it themselves, because they do not trust the data. We noticed this early on and conducted many interviews, also with other companies. The issue of trust was always a problem. That is why we chose the approach to identify and involve suitable employees from the beginning. They then believe that they built the system and that it works very well. However, the management using the model also pushes this attitude.

To summarize, the top management team is pushing for using data analytics and provides departments with capabilities (data scientists). It is within each department that the projects are developed and under the supervision of controllers who decide on the needs. The project leader was also asked about how he set his team up, whom he selected, and whom he had integrated. Although he is a data scientist, the accounting and finance department employed him. Consequently, he surrounded himself with controllers who could help him steer the project. The top management team supported them strongly, and the project was a great success.

### InduCo

InduCo is a global industrial company based in Europe. It produces and develops electrification products, components for power grids, and industrial automation and robotics components. Its yearly sales income is almost EUR 30 billion. Our interlocutor was a Lead Data Scientist involved in designing the project’s technical solution. It is essential for an industrial company to accurately forecast order entries in order to optimize its supply chain operations and take proactive measures regarding its financial control. Consequently, as the Lead Data Scientist described, InduCo decided to digitalize its hitherto manual forecast process by means of predictive analytics:

At that time, the forecasting was a manual process carried out very elaborately and involving 1,600 people every month. It was also not very accurate. Since this was a pain point, involved a lot of people, and was too inaccurate, we wanted to do it differently.

The interviewed data scientist explained that the project was mostly focused on financial and internal data:

Interviewer: “One step before the data mining and with regard to the data cleansing and transformation. How much effort did it require for you to prepare the data?”

Interviewee: “Actually, no effort with respect to the internal data because we received the data directly from the data warehouse and they were all compatible. It also required little effort in respect of the external data, as the problem was rather how we to match the Bloomberg and the internal data. However, all the [internal] data are very well maintained, which means we pay for the data’s quality.

The initial plan was to include nonfinancial and qualitative data, but this plan could not be carried out because the data’s quality was insufficient:

The data were unfortunately incomplete. This (i.e., commenting orders) has been done in a few countries, but most [employees] do not comment because they do not have to. Further, it was questionable whether we could extract relevant factors from the limited available data.

The biggest challenge that InduCo faced was in terms of the organizational structure:

The biggest challenges were not technical, just organizational. Receiving feedback, or buy-in, or time from the divisional controllers was always a struggle and required a great deal of time.

Convincing the various stakeholders took a great deal of time:

I think we spent a lot of time collecting and integrating the stakeholders, also in terms of the planning and the development. However, we have the feeling that they were actually not as confident as they said they were (…). I think was because they did not understand the process, as the background is just comprised of mathematics and not guessing. The entire organization then halted the process.

Despite a significant effort, the data scientists could not manage to create a convergence between all the actors. However, One of the scientists also gave some hints that he was not leading the project in all dimensions. For example, he could not judge whether the system was used in practice:

That’s difficult for me to judge. It was the division controller to ensure that this was used in the division.

The project was not InduCo’s main priority:

The team was already busy, and they only did the minimum that was required, and the project team also took care of this.

The lack of internal analytical knowledge might partially explain the overall lack of trust, understanding, and willingness to participate in the project. Efforts were required to build a successful team with an appropriate profile balance:

As a data specialist, I had an external data scientist who was familiar with SAP Tools. In addition, we had two economists with some data science skills, but they also undertook the macroeconomic analysis, while there was someone to develop the front-end.

The arrival of external partners might also have increased the lack of trust in the project, which increased when the project was implemented. The implementation impacted the organization; changes should therefore be monitored, which would avoid resistance to projects like those we sometimes observed. The interviewed data scientist explained that they failed to involve the top management:

If there is too little top management support, the project will simply not be implemented.

Even after the system had gone live, nobody wanted to take responsibilities for driving the changes. Consequently, the project suffered a lack of clear leadership in respect of the employees:

This [leading the use of the system] was the task of the divisional controller. It seems that once the solution went live, they [the users] no longer did anything.

Ultimately, this resulted in the project being abandoned:

There was no more budget for running it in the organization. Nobody was willing to pay for the system’s maintenance. Our unit was unfortunately also dissolved.

## Findings

This section presents the findings regarding the presented cases. Two main contributions are made. The first one concerns the role of the controller. The observations show that without a controller steering the project, it was likely to fail, even when its value-added could be assessed. Second, we present the three project trajectories we could observe and design a framework presenting the trajectories.

### Role of controllers

Six data analysis projects in financial function are observed. In the six projects, the changes being implemented concerned at least one controller (part of the finance department). We examined the role controllers played and how important the results of this role were. Sometimes the relevant controller was the case interviewee. At other times a case interviewee discussed his interaction with a controller and the role this controller fulfilled. This could be a limitation of this study, as people might shift the responsibility for mistakes to other parties.

Observations showed that the controller played a central role in data analytics projects related to business forecasting. He acted as the catalyst between up to three types of partners: the top management, more precisely the CEO or the CFO; the technical solution implementers, such as data scientists from within the organization or as external partners, and users. The controllers in the six projects were sometimes also part of the user group but, in most cases, were not the only ones. Other controllers, accountants, and potentially also salespeople and other people were directly involved in the business transactions impacted by the new solutions. Other potential stakeholders are not excluded but were not observed here.

In all the investigated cases, the trust created in the project was a very interesting point. Each of the three types of stakeholders described above had to trust and believe in the project for it to work. The top management had to be convinced of the financial profit that could be realized. On the other hand, the implementors needed to understand how they could simplify the work of others. Finally, the user had to be convinced that the project was going to facilitate their daily work. If the data analytics projects were to simplify the operations, the transition to the new system and processes could have a significant cost (in terms of time, finance, new practices to be learned, new software to be developed, etc.). Consequently, the stakeholders needed to foresee a long-term benefit in order to be willing to pay the short-term transition cost. The need to generate trust in the project aligns with prior literature (Antoniadis et al., 2015; Klatt et al., 2011; Ram et al., 2014). In the six specific cases here, however, the role of the controller appeared to be more prominent than that of other stakeholders such as top management (Bansal & Agarwal, 2015; Morelli & Lecci, 2014) or external partners (Antoniadis et al., 2015; Bansal & Agarwal, 2015).

Controllers’ roles could have a significant impact on a project’s success or failure. The six projects concerned, at least in part, the use of financial data for enhancing the quality, accuracy, or availability of forecasts. Often, the need arose in the controlling department. At ChemCo, TechCo, and TelCo, the projects were initiated by a controller. Consequently, they took a leadership role in the project. Their first role was to convince the top management of the need for the project. Contrary to previous literature on information systems implementation, the initial impetus mostly came from controllers rather than top management (Bansal & Agarwal, 2015). If the limited number of cases could explain the divergence from previous contributions, the specific aspect of forecasting activities, for which the controller is critically involved, might also be an explanatory factor that should be further investigated. Once the controller initiated the project, two main patterns were observed. First, strong support, which was the case at SoftCo and ChemCo. Since the controller obtained the top management’s support rather easily, the project subsequently ran smoothly. It should be mentioned that the project leader at SoftCo was a data scientist who the finance and accounting department employed. Although not a controller, he put a great deal of effort into integrating the users’ perspective by simplifying their work.

In some of the cases, the top management only gave a mild support to the project team. As it turned out, the projects were not directly discarded but the project owners were allowed (or required) to keep it running in parallel to the standard system and processes or to develop a test project. At that moment, the controllers in the cases studied evolved in two different ways. They either regarded the mild support as a challenge to solve or tried to avoid addressing it. Interestingly, controllers took a central role at that point. If they wanted to lead, they had a chance to convince others. They could mostly achieve this by proposing a slow path of gradual wins. This happened at TechCo and FoodCo. The management was not convinced of their projects’ merit but allowed them to carry on. As leading controllers, they created a path toward the final goal they wanted to achieve, which they sold over time while successfully achieving each milestone they set. The opportunity to keep a project going even with mild top management support, as observed in these cases, is somewhat contradictory to previous research (Morelli & Lecci, 2014; Raafat Saade & Nijherhors, 2016). It might be an indicator that, unlike other information systems and technologies, data analytics could affect controlling differently (Appelbaum et al., 2017; Quattrone, 2016; Vasarhelyi et al., 2015). The limited number of cases studied do not allow for generalization, but rather call for more investigation in larger samples.

At TelCo, the controllers did not have such a plan. They failed to steer the overall project, and, after some time, their budgets were cut. However, the question is whether intermediate results and milestones down the road might have change the outcome of the project. Nevertheless, the lack of agile implementation was probably not the only killer. Ultimately, nobody volunteered to run the implemented system, no one in the controlling department wanted to lead and accept responsibility, and the project was dropped.

A group of data scientists at InduCo led the project. They discussed the top management’s lack of support, but neither the controllers nor the users were sufficiently integrated into the project to take the lead. The trust path they foresaw was for them to convince the top management, who would then be responsible for integrating the users. As this observation only represents one example, it would be impossible to conclude a causality, still, the group of controllers was never put in the leading position (Wolf et al., 2020) and the project ultimately failed. Interviews showed the incapability of generating trust through a centrally placed controller, where in other cases, trust towards the controller was identified as a success factor.

At ChemCo, even if job cuts were set as a target, the controlling department employees led the project. In this particular case, the leading controller played a central role in creating trust and was the best of the three groups of stakeholders at steering the project and generate a coalition around the goal (Chua et al., 2012). In the cases observed, the presence of a leading controller driving the project well and convincing the top management of the need for such technologies seemed to produce better results in term of implementation. The opposite was also observed. In the absence of a leading controller driving the project. The timing of the failure is difficult to clearly define, given the limited number of cases studied. Observations showed that the failure could arise quite fast (preventing the project from starting) or towards the end, when the developed solution is about to go live. Controllers were not often pushed by other stakeholders towards being the drivers of the digitalization of practices. They were also not used to leading cross-departmental projects. Controllers are used to play a supporting role but are advised to take wider role of business partners in the future (Goretzki et al., 2013). We are heading toward a world in which the development and implementation of new complex control systems (of which data analytics is one) require controllers to take a more extensive role. In other information systems projects, such as ERP implementation, the controller was mostly kept in the shadow by top management and external consultants (Raafat Saade & Nijherhors, 2016). Convincing, leading, and integrating stakeholders were not characteristics expected from controllers in the past. However, advanced data technology could open a new field for controllers; if they manage to develop the required competencies, they will be the best located to accept these new responsibilities. The leadership role that controllers can take in leading data analytics projects appeared in the cases studied to be a critical success factor.

### Strategy implementation framework

**Fig. 1:**
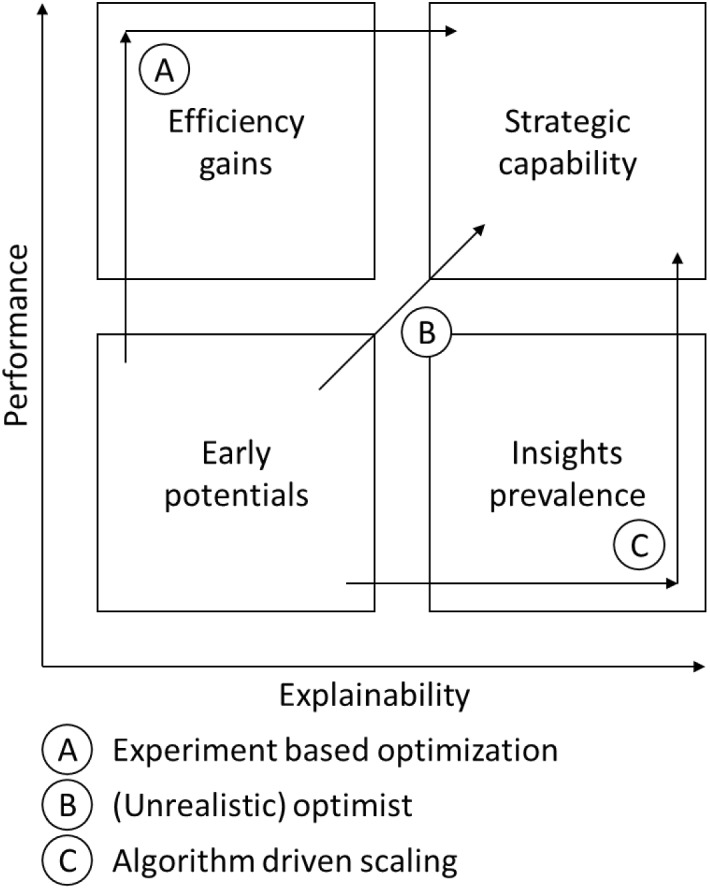
Strategic Implementation Model

When analyzing the projects’ initial objectives, how these were defined and their ultimate results, we identified three implementation strategies, as shown in Fig. 1. In the six cases studied, the companies realized that their data had potential and managed to create early wins. On the one hand, data analytics was subsequently used to make these companies either more efficient (using fewer resources) or to allow them to obtain similar information or outcomes (Bergmann et al., 2020; Knauer et al., 2020; Perkhofer et al., 2020; Vitale et al., 2020). Companies could therefore increase the efficiency or *performance* of their forecasting. However, on the other hand, data analytics could help companies gain better information or new business insights. In this case, companies progressed on the explainability scale, which allows them to extract more valuable information on how to drive their firm performance (Appelbaum et al., 2017; Roßmann et al., 2018; Wang et al., 2018; Wilson, 2018). Combining the two data analytics results allowed the companies to use data analytics as a strategic tool to, simultaneously, not only gain a new competitive advantage but also become more efficient.

We plotted the strategies we had observed in this graph. One of the strategies (B) was that of very ambitious teams who wanted to increase the precision of their predictions or the insights that the data analytics provided while, at the same time, reducing the resources they used. TelCo and InduCo were in this category, which, unfortunately, turned out badly for both. While the likelihood that their strategy might have been successfully implemented should not be ignored, one should also ask whether being too ambitious and optimistic undermined the overall project.

In respect of InduCo, it was unclear whether the project was initiated to eventually provide accurate and usable predictions and, later, realize efficiency gains or whether the objectives were reversed. In other words, since the system did not provide accurate results, the number of resources dedicated to it was reduced. The Lead Data scientist did not understand the sequence of the events. A project with clear sequential events might have been more successful, as was observed in the four other cases.

Another identified implementation strategy was that of ChemCo and SoftCo (A). Both started their respective project to reduce costs or, more generally, the resources consumed. While succeeding in their initial objective, they subsequently realized they could also gain precision and insights with their new process. Once they had a successful process, some experimentation took place. They realized that data analytics could help them do a better job and not just do their previous job more efficiently. Their system was therefore adapted to fit this new objective. This process also arguably produced the most successful pattern in the sample of six cases. The explanation for this success could be that when the metrics showed the system’s success, the employees were convinced and supported the further development of the data analytics project.

FoodCo and TechCo (C) used the final identified implementation strategy. They started their projects because they required better information. The forecasting systems they used were not good enough to provide the businesses with the information they needed to grow, identify, and capture new markets. In this situation where the business continuity was at stake, the resources consumed to gain better forecasts were not the major concern for the management. They felt that once their projects were successfully deployed, they could potentially save resources. However, this could only succeed if they rearranged their new systems’ processes and organizations.

The clarity of the pursued goal appears to be critical for the success of a data analytics project (Appelbaum et al., 2017; Yeoh & Koronios, 2010), but it is not always mentioned as one for other types of information systems projects (Raafat Saade & Nijherhors, 2016). The project’s success being dependent on the clarity of its goal might sound tautological for a system implementation for which the outputs are clearly evaluated. For data analytics, however, measuring the impact of the technology and the changes it can imply is more complex. It is aligned with the current debate on the role that will play data analytics in controlling (Bhimani & Willcocks, 2014; Griffin & Wright, 2015; Quattrone, 2016). Our observations indicate that the technology’s added value is so vast that its full impact cannot yet be measured.

The case studies helped to identify two major critical success factors that differ from other information systems projects. The relationship between these two factors is also of interest (Bansal & Agarwal, 2015). The leadership of the controller over the project correlated with a better goal definition and planning. If, for some information systems, the top management team can have a better understanding of opportunities and needs, current results suggest that the operating side of control can have a more significant role to play in assessing the potential of data analytics. Such observations suggest that data analytics, unlike other technologies, have the potential to radically change the roles and responsibilities of the controller and the controlling departments (Appelbaum et al., 2017; Bhimani & Willcocks, 2014; Griffin & Wright, 2015; Vasarhelyi et al., 2015) and call for a more in-depth investigation of the phenomenon.

## Contributions

The literature highlighted the need for further empirical research on the impact of data analytics on management accounting (Gärtner & Hiebl, 2017; Rikhardsson & Yigitbasioglu, 2018). The cases confirmed that need by shedding light on aspects not yet adequately documented but which seemed to be of central importance. The most interesting of these aspects was the challenge to generate trust in the project. The case study approach showed that trust might depend on various factors. At the same time, the specificities of the forecasting focus could be a catalyzer for trust issues, with both issues calling for a better study of trust. Our study also confirmed that integrating predictive analytics into the business performance forecasting process could result in three major potential gains: accuracy, improved business insights, and improved efficiency (Roßmann et al., 2018; Wang et al., 2018; Wilson, 2018).

Taking a qualitative approach rather than more quantitative ones (Bergmann et al., 2020) allowed us to observe similarities in successful projects. For example, the need for sound data systems before developing a data analytics project and the value-add of already being a technology-versed company. Our results, however, indicate that a linear correlation might not explain a project’s success or failure.

Generally, the cases investigated provided highly accurate results. Interestingly, they achieved this accuracy by means of a broad range of methods. All the cases experimented with various statistical methods and some even with additional data analytics techniques, such as machine learning components. One could conclude that no single generic pattern allowed the investigated companies to achieve accurate predictive results, but their success depended on the case goals and the available data (Otley, 2016).

Predictive analytics also holds the promise of providing business insights. In fact, scholars believe that generally engaging with the underlying data in such projects could lead to a better understanding of genuine business drivers (Chase, 2014). However, only a few of the cases studied could achieve this goal and then only to a certain extent. An explorative dashboard displaying the indicators with the highest impacts on the predictions (e.g., at ChemCo and InduCo) appeared to be the most promising tool among the cases studied. TelCo also wanted to obtain enhanced knowledge of business drivers but failed to transform the operational data into (traditional) financial KPIs.

The gain in efficiency, which was only possible after the project’s successful integration into the operational processes, was another observed result. However, two aspects of efficiency need to be distinguished: first, the amount of time required to create a forecast and, second, the number of resources required. Regarding the required time, all the cases implemented a highly automated solution, which meant that they could quickly create a forecast. Nevertheless, only SoftCo could exploit the efficiency gains by completely replacing its manual forecast, while ChemCo planned to do so in 2019 (Holland & Light, 1999).

The current paper helped to understand better the role played by controllers in the specific cases of using data analytics. In the cases studied, the capability of the controller to take the leadership role in the data analytics projects was associated with success in the project (Goretzki et al., 2013). If the limited number of cases do not allow for wider generalization, it is arguable that, in the future, controllers will be increasingly faced with a situation in which they have to take a leading role in implementing data analytics-driven projects. Their position within the organization will also change, as they will have to leave their department, become involved, and lead cross-departmental projects. Does the need to place the controller in charge of the project stem from the nature of data analytics, or is it the consequence of another factor? These results contribute to the debate on the controller’s future role in integrating these new technologies. The observations call for a better investigation of why data analytics is not simply a new system like ERP was (Alles, 2015; Quattrone, 2016).

The interaction between the need to specify the objective pursued with data analytics and the knowledge the controller can mobilize when he is leading the project calls for more investigation of the relationship. It is likely that this reinforces his future role in the leadership of the organization and the potentially radical changes inferred by data analytics on controlling practices.

In summary, our observations showed that companies manage to leverage on data analytics to achieve gains (efficiency, precision, new insights) which confirmed what conceptual papers perceived in the context of management accounting and, more specifically, business forecasting. In the cases studied, however, we also observed that pursuing multiple goals at ones (including gain of efficiency) might lead to the failure of the project.

Our research also identified some direction for how and where to study the future roles of accountants. Broadly, accountants could either extend their competencies toward understanding and developing technology or the managerial aspects linked to technology implementation. Our findings tend to favor the second option.

## Limitations, future research, and conclusion

As previously mentioned, the limited number of interviews and, especially, interviewing only one person per company means that the information captured might reflect or be biased toward that person’s personal view. It might not entirely reflect the organizational perspective.

Besides the above, another limitation concerns the dimensions studied in the cases. These dimensions influenced and limited what could be identified and where these factors could be identified in the organization.

The approach followed, which derived general rules from specific cases could be seen as flawed as a result of the above limitations. The contributions provide propositions that could be further tested in a wider research setup. Our findings depended strongly on the selected cases. The findings contain similarities with prior research. The current research identified two success factors that seem to play a more prominent role in the specific case of data analytics.

Another limitation is the emerging trend to base data analytics for business performance forecasting on unstructured data. If some attempts to use such data are made, the selected case studies only used structured data. Some of the cases did consider adding qualitative data but did not yet achieve success. Practically, it would be challenging to identify case studies with already implemented such use of their data. Consequently, the findings do not necessarily apply to implemented projects based on unstructured data.

From a theoretical perspective, this paper contributes to the debate on the potential radical impact of data analytics on controlling functions. Based on exploratory techniques, the observations provided an initial reading grid for further research in the field. It also validated previously developed concepts for studying performance management and data analytics forecasting regarding information systems implementation.

From a practical perspective, the contributions give managers tools to prepare for the implementation of a digitalization project. They can, at least, serve as a tool for project managers to reflect on and, at best, as guiding principles for the overall project development and implementation. Such tools could also help analyze existing but imperfect projects, or even failed ones, or those about to fail, to improve the situation and get it back on track.

## Interview guideline

1. Context
2. Can you summarize in a few sentences what your forecast is based on and what it is used for in your company?
3. What exactly is forecasted? Individual line items or the entire income statement? Which granularity is forecast? What time horizon is predicted?
4. When was implemented the Forecast in its current form and are there any plans to expand or adjust it?
5. Data
6. What data do you use as the basis for data mining? (external / internal, structured / unstructured)
7. How far do you go back in history of the data you are using?
8. Are there any plans to include further data (e.g., external / unstructured data)?
9. If yes, why are they not being used yet?
10. Methods
11. What data mining techniques do you use in forecasting (algorithms / groups)?
12. How many variables do you use in your model?
13. Do you use machine learning components and/or neural networks?
14. If No, why not? Are there any plans to introduce them?
15. Do you use value drivers to aggregate individual line items?
16. How much effort do you spend on cleansing and transforming data before you can use data mining techniques? What exactly is being done?
17. Are the results of the forecast visualizable for the user? How is this done?
18. Impact
19. To what extent are the results of the forecast integrated into the operational controlling process? Are operational decisions made based on this?
20. Can you say something about efficiency or resource savings compared to the previous process?
21. How accurate are the results of the forecast compared to alternative manual forecasts?
22. Do people trust the results of the forecast in your company or are you challenged by certain stakeholders?
23. If yes, why do you think this is so?
24. Organization & IT system
25. How is the Forecast process organized in your company?
26. Is there a dedicated ‘Analytics’ department? With specific skills such as Data Scientist?
27. What kind of tasks and projects is this department doing exactly?
28. Do you have a standardized process in your company to identify and implement analytics use cases?
29. What software components do you use in your forecasting process?
30. How are these integrated into your overall IT landscape? (e.g., central data lake, data warehouse, interface layer, ERP, reporting)
31. Is your IT landscape ready to process big data? If no, are there plans to make changes in this area?
32. Implementation
33. In which project steps was your forecast implemented? (i.e., prototype phase)
34. Was there top management team support?
35. What approach was used to implement this project? (waterfall, Scrum/Agile)
36. What change management measures were taken?
37. Did you have external support to implement the project? What was it? (Developers, IT & Business Consultants)
38. What were the biggest challenges in the project?

## Footnotes

1 The term ‘big data’ is used here in conjunction with the decision-making it is used for. Consequently, it is in line with definitions provided above and on which a consensus has been formed.

2 International Data Group. 2018. Predictive Analytics 2018., E-skills UK. 2013. Big data analytics: adoption and employment trends, 2012–2017.
